# Characteristics of malignant tumors of digestive system and autoimmune diseases

**DOI:** 10.3389/fgene.2023.1147047

**Published:** 2023-03-16

**Authors:** Hang Yin, Qun Li

**Affiliations:** ^1^ Day Clinic, The Hainan Hospital of Chinese PLA General Hospital, Sanya, Hainan, China; ^2^ Department of Thoracic Surgery, The Hainan Hospital of Chinese PLA General Hospital, Sanya, Hainan, China

**Keywords:** digestive system, malignant tumor, characteristics analysis, D-dimer, autoimmune diseases

## Abstract

With the development of preoperative examination technology, endoscopic ultrasonography-guided fine-needle aspiration biopsy (EUS-FNA) has been widely used in preoperative pathological diagnosis. However, challenges remain in obtaining appropriate tissue samples and obtaining accurate pathological results to predict disease risk. Therefore, this study aimed to analyze the characteristics of digestive system malignancies and their autoimmune diseases, and to analyze the clinicopathological features, preoperative CT imaging features and pathological grades of pNENs of different pathological degrees of pNENs on the prognosis of pNENs. Experimental results showed that non-functioning pancreatic neuroendocrine tumors showed prominent surrounding hypervascular lesions on multiphase CT examination. Among them, arterial phase and portal venous phase were most clearly imaged at the end, and the degree of local vascular invasion could be used as an index to evaluate its resectability. The sensitivity of CT examination was 63% to 82%, and the specificity was 83% to 100%, depending on the size.

## 1 Introduction

Currently, Stroke has been found to be the most common neurological complication in cancer patients other than tumor brain metastases. It has been reported that up to about 15% of cancer patient autopsies show thromboembolic complications, and 50% of these patients have clinical manifestations of stroke during their lifetime. Retrospective studies have shown that malignancy is diagnosed in 6%–12% of patients with ischemic stroke. In actual clinical work, however, these numbers are much smaller because the association between stroke and cancer is often overlooked by clinicians. This is often because cancer-related strokes are difficult to diagnose, especially in patients with no known cancer history or strokes of unknown origin. Patients with cancer may experience a stroke in a different way than others without the disease. It is well accepted that blood hypercoagulability caused by cancer plays a major role in the underlying mechanism of stroke in cancer patients. At present, there are more and more relevant studies, but the sample size of the studies is generally small, and there is still a lack of systematic studies with large sample sizes.

The survival rate of cancer patients is rising, and more cancer patients are also developing strokes, thanks to the quick development of more efficient cancer treatments. Therefore, research into the connection between cancer and acute ischemic stroke is crucial. In patients with malignant tumors complicated by acute ischemic cerebral infarction, this study conducted a retrospective case-control investigation on the clinical characteristics, vascular risk factors, and imaging characteristics of conventional stroke. This article discusses the related risk factors of CAIS, aiming to improve the clinical understanding of the disease and provide a theoretical basis for clinical diagnosis and treatment.

The innovation point of this study: 1) The CT imaging data (including outpatient and inpatient CT imaging data) of 83 patients were investigated. This article retrospectively analyzed preoperative CT images and pNEN classification. And this paper compared the differences in CT imaging features between G1 and G2. 2) the data of 56 patients were examined and analyzed to obtain CT values of arterial phase, portal venous phase, advanced tumors and normal tissues, as well as CT imaging features of G1pNEN and G2pNEN patients. In this paper, the data of 56 patients were examined and analyzed to obtain the CT values of arterial phase, portal venous phase, advanced tumor and normal tissues, as well as the CT imaging characteristics of G1pNEN and G2pNEN patients. Thus, the difference and ROC curve were compared to analyze the diagnostic value of absolute and relative CT magnification of tumor lesions in distinguishing G1 and G2 patients.

## 2 Related work

Numerous autoimmune disorders seem to affect women more often, which raises the possibility of a hormonal cause. There are minimal research on tumor estrogen receptor (ER) and progesterone receptor (PR) status, as well as breast cancer risk in systemic and organ-specific autoimmune disorders. Schairer evaluated the relationship between several organ- and system-specific autoimmune disorders, as well as tumor ER and PR status, and overall breast cancer risk in this study. They discovered the first case of breast cancer in a woman under the age of 66 using Linked Surveillance, Epidemiology and End Results (SEER)-Medicare data. They used unconditional and multinomial logistic regression to estimate odds ratios (ORs) and 99.9% confidence intervals (CIs) (Schairer et al., 2018). Immune checkpoint inhibitors (ICIs) are being used more frequently in the metastatic situation or as a supplement to other therapies to treat a wide variety of cancer types. Due to worries about increased toxicity, patients with autoimmune illnesses have been mostly excluded from clinical trials utilizing ICIs. Kennedy et al. (2019) LC reviews current case series describing patients with solid tumors who already have autoimmune disease and examines similarities between clinical autoimmune illness and ICI-induced immunotherapy-related side effects. Which autoimmune patients are likely candidates for ICI therapy is also included in his review (Kennedy et al., 2019). However, no trials have examined its usage, either as a monotherapy or as a combination medication, in the treatment of cancer. According to the Hong J study, Y-320 caused significant harm to MDR tumor cells when it was given concurrently with other chemotherapeutic drugs, although having little effect on multidrug-resistant (MDR) cell lines. Further investigation indicated that P-glycoprotein is a substrate of Y-320 (P-gp). When paclitaxel and Y-320 were administered together, tumor development was dramatically suppressed by 77.1%, as opposed to 6.5% in the group receiving paclitaxel alone (Hong et al., 2020). Numerous autoimmune disorders seem to affect women more often, which raises the possibility of a hormonal cause. Kennedy LC evaluated the relationship between several systemic and organ-specific autoimmune disorders, overall breast cancer risk, and tumor ER and PR status in this study. Despite the fact that these samples were Medicare recipients in good health and free of breast cancer, there was some experimental risk (Kennedy et al., 2019). Immune checkpoint inhibitors (ICIs) are being used more frequently in the metastatic situation or as a supplement to other therapies to treat a wide variety of cancer types. The similarities between clinical autoimmune disorders and the unfavorable effects of ICI-induced immunotherapy are discussed by Radovich et al. (2018). Additionally, he reviews case studies that depict individuals who have solid tumors and pre-existing autoimmune disorders. The prevalence of autoimmune reactions brought on by immune checkpoint blockage during cancer treatment emphasizes the value of interdisciplinary research on autoimmune illnesses and malignancies. In lymphoid malignancies, Shao et al. (2019) points out the connection between particular IL-7R signaling and steroid resistance. Additionally, he showed that after receiving steroid treatment, IL-7R-positive cells that are resistant to steroids may be found in mouse bone marrow, spleen, or in a mouse model of autoimmune arthritis. Everolimus, a mTOR inhibitor, may be used in patients with advanced relapsed and refractory thymic epithelial malignancies, according to newly available research (TET). The identification of patients and the discovery of predictive biomarkers of response are still difficult tasks. Here, Yan et al. (2017) gives a complete genetic characterization of TET patients’ thymic tumors and describes a single-center experience with everolimus in those patients. There were still mistakes in the calculations of time to treatment failure and overall survival even though patients had had significant pretreatment with an average of three prior lines of treatment.

## 3 Medical image enhancement algorithms

### 3.1 Histogram equalization

The histogram equalization method has a relatively simple theoretical principle, a short running time and the maximum possible preservation of the original image information. It is often used in medical image enhancement (Hellyer et al., 2020). This method remaps the pixel value distribution range of the image to the entire gray level range to enhance the image contrast.

Histogram equalization (HE) expands the gray-level distribution range of the original input image by counting the pixel values, distribute the original image histogram to the global gray level range through the remapping function [the gray level range of the 8-bit image is in (0,255)], and avoids the concentration of pixel values in a certain gray-scale range (AlKindi, 2017; Li et al., 2017). The original input image is defined as X, and its probability density distribution function is shown in Formula (1);

$$p\left(X_{k}\right)=\frac{n_{k}}{n}$$ (1)

$$c_{\left(x\right)}=\sum_{i=0}^{k}p\left(X_{i}\right)$$ (2)

Among them, x = $X_{k}$ , k=0,1,…,L−1 , it can be deduced that c (L-1) = 1. Histogram equalization is to perform a function transformation operation on the pixel distribution histogram of the image. The process is to stretch and redistribute the histogram distribution of the input image through a certain function transformation (Nozaka et al., 2020; Ohkubo et al., 2020). For an image with a dynamic range of ( $X_{k}$ ; $X_{L-1}$ ), its mapping function can be defined as:

$$f_{\left(x\right)}=X_{0}+\left(X_{0}+X_{L-1}\right)c_{\left(x\right)}$$ (3)

The advantages of the histogram equalization algorithm are mainly reflected in that it regards the histogram of the input image as a whole operation unit, performs global mapping function f(x) transformation on the image pixels, and expands the distribution range of dynamic gray levels (Agra et al., 2020; Ryabov et al., 2020). In addition, according to the information entropy theory, it is easy to know that when the probability distribution of pixel values is uniform, the value of image information entropy is the largest, which makes the enhanced image outline clear and colorful (Zhao et al., 2020).

### 3.2 Bipartite histogram equalization

The bisection histogram equalization algorithm first calculates the gray mean value of the image, and then divides the histogram of the original image into two parts according to this value. Finally, the two sub-histograms are equalized respectively, so as to better maintain the original brightness of the image. Let $X_{m}$ be the mean of the image X, from which we know $X_{m}\in$ ( $X_{0},X_{1},\ldots ,X_{L-1}$ ). Taking the image brightness mean point $X_{m}$ as the boundary, the histogram is divided into two parts: $X_{L}$ , and $X_{U}$ (Lukianchenko et al., 2020). The dynamic range of the pixels in the lower half of the histogram is set to $X_{L}\in$ ( $X_{0},X_{1},\ldots ,X_{m}$ ). The dynamic range of the pixels in the upper half of the histogram is in $X_{U}\in$ ( $X_{m+1},X_{m+2},\ldots \;X_{L-1}$ ), and their respective probability density distribution functions are shown in Formula (4) and Formula (5), and the expressions are:

$$p_{L}\left(X_{k}\right)=\frac{n_{L}^{k}}{n_{L}}$$ (4)

$$p_{U}\left(X_{k}\right)=\frac{n_{U}^{k}}{n_{U}}$$ (5)

Among them, the value of the density distribution function k of the upper and lower parts is taken as k=0,1,…m and k=m+1,m+2,…L−1 respectively. $n_{L}$ represents the sum of pixels in the lower half $n_{L}$ = $\sum_{k=0}^{m}n_{l}^{k}$ , and $n_{U}$ represents the sum of pixels in the upper half $n_{U}=\sum_{k=m+1}^{L-1}n_{u}^{k}$ . It can be seen that n = $n_{L}$ + $n_{U}$ .

According to the conditions, the average brightness of the image enhanced by the He algorithm is equal to the median of the pixel display range (Li et al., 2020; Makhdoomi et al., 2020). Therefore, such methods are not applicable in many fields. The average brightness of the image enhanced by the BBHE algorithm will be limited by the average brightness of the histogram of the high and low parts, thus playing the role of brightness preservation.

### 3.3 Histogram equalization based on motion blur

The histogram equalization method based on dynamic blur (BPDFHE) can improve the problem of detail loss to a certain extent (Manikandan, 2020). The dynamic blur histogram equalization method mainly uses the process of fuzzy statistics to map the input image to the fuzzy domain through the transformation function. This method effectively solves the roughness of the grayscale range of the image, which is beneficial to improve the performance of the algorithm.

First, the input image histogram needs to be transformed into a blur histogram through a function. A fuzzy histogram is a sequence of real numbers consisting of h(X) $X\in \left\{0,1,\ldots ,L-1\right\}$ , where h(X) represents the distribution probability of gray levels in the neighborhood of X. $\tilde{I}$ (i,j) is set as the grayscale blur value of I (i,j), and the blur histogram is shown in Formula (6):

$$h\left(X\right)\leftarrow h\left(X\right)+\underset{i}{\sum }\underset{j}{\sum }\mu_{\left(i,j\right)X},k\in \left[a,b\right]$$ (6)

Among them, $\mu_{I\left(i,j\right)i}$ is a triangular fuzzy membership function. Next, take the local maximum value of the histogram as the limit point to obtain a plurality of sub-histograms. Denote the first derivative and second derivative of the fuzzy histogram as $h^{\prime }\left(X\right)$ , h"X), respectively, and their derivative expressions are:

$$h^{\prime }\left(X\right)=\frac{dh\left(X\right)}{dX}=\frac{h\left(X+1\right)-h\left(X-1\right)}{2}$$ (7)

$$h^{\prime \prime }\left(X\right)=\frac{d^{2}h\left(X\right)}{dX^{2}}=h\left(X+1\right)-2h\left(X\right)+h\left(X-1\right)$$ (8)

The second derivative h”(X) directly uses the second central difference operator to calculate the second derivative of the fuzzy histogram. The purpose of this is to minimize the approximation error accumulated from the first derivative calculation. After the histogram is divided into multiple sub-histograms, each sub-histogram is remapped to the gray level dynamic range, and finally the brightness of the image is normalized.

$$\frac{E}{S}=T\left(\frac{p2}{p1}\right)*T\left(\frac{p3}{p2}\right)*,,,*T\left(\frac{pn}{pn-1}\right)$$ (9)

$$\log\left(\frac{E}{S}\right)=\log\left[T\left(\frac{p2}{p1}\right)\right]+\log\left(T\left(\frac{p3}{p2}\right)\right)+,,,+\log\left(T\left(\frac{pn}{p_{n-1}}\right)\right)=\sum_{i=2}^{n}\log\left[\left(T\left(\frac{pi}{p_{i-1}}\right)\right)\right]$$ (10)

$$\log\left(\frac{E}{S}\right)=\sum_{i=2}^{n}\log\left[\left(T^{\prime }\left(\frac{pi}{p_{i-1}}\right)\right)\right]=\overset{n}{\underset{i=2}{\sum }}T^{\prime }\left(\log p_{i}-\log p_{i-1}\right)$$ (11)

$$D=2^{\left.\log2\min\left(rows,cols\right)-1\right)}$$ (12)

After one comparison, rotate 90° clockwise, the distance is halved, convert Maiko D = -D/2 and continue the comparison until |p|<1. The iterative Formula is as follows:

$$r_{n+1}\left(x,y\right)=\frac{r_{n}\left(x,y\right)+r_{n}^{\prime }\left(x,y\right)}{2}$$ (13)

$$r_{n}^{\prime }\left(x,y\right)=\left\{\begin{array}{c} r_{n}\left(x,y\right)+∆L\;r_{n}\left(x,y\right)+∆L\leq \max\\ \max\;r_{n}\left(x,y\right)+∆L\leq \max \end{array}\right.$$ (14)

The single-scale Retinex algorithm expression is

$$logR\left(x,y\right)=logI\left(x,y\right)-\log\left(F\left(x,y\right)*I\left(x,y\right)\right)$$ (15)

$$F\left(x,y\right)=K\cdot e^{\frac{-\left(x^{2}+y^{2}\right)}{\sigma^{2}}}$$ (16)

$$\iint F\left(x,y\right)dxdy=1$$ (17)

## 4 Experimental characteristics

Through previous studies, we found that in the comparison of the pathological characteristics of GI grade and G2 grade pNENs, G2 grade pNENs and patients’ tumors were larger in maximum diameter, more prone to invasive growth, more prone to lymph node metastasis, and more prone to distant metastasis. Preoperative invasive EUS + FNA examination cannot obtain 100% accurate pathological results. So whether these differences can be reflected by simple and easy CT imaging examination, we combined CT, the most commonly used preoperative examination method in 266 patients, to screen out 83 cases of G1 and G2 grades with CT images. The CT imaging features were used to analyze the differences in imaging features between G1 and G2 patients, and to observe whether the pathological grade of the patients could be judged by CT imaging. Through the further quantified CT indexes of 56 patients with pancreatic neuroendocrine tumors, we measured the CT values of arterial phase, delayed phase, portal venous phase, plain scan tumor and normal tissue. To analyze the significance of CT imaging features on the pathological grading of pancreatic neuroendocrine tumors, so as to predict its possible prognosis and then adopt more appropriate surgical methods.

### 4.1 Materials and methods

#### 4.1.1 Research objects

Inclusion criteria for patients with acute cerebral infarction with active cancer (cancer group, *n* = 28) were as follows. 1) Acute ischemic cerebral infarction is defined as sudden focal or generalized neurological damage that persists for >24 h and has imaging-related symptoms, or symptoms of neurological damage <24 h. However, the patient’s acute ischemic cerebral infarction was verified by MRI diffusion-weighted imaging (DWI). 2) Active cancer was defined as a malignant tumor that had been diagnosed or untreated within 1 year before the stroke, as confirmed by the patient’s previous medical records. Either a previously inactive malignancy that recurred within 1 year before the stroke, or a newly diagnosed malignancy during the stroke hospitalization. The diagnosis of tumor is confirmed by tissue or liquid pathological biopsy. Inclusion standards for the cancer group were as follows: 1) Patients without diffusion-weighted imaging. 2) Patients with clinically diagnosed tumors but not confirmed by pathological biopsy; 3) Patients with benign tumors, secondary intracranial metastatic tumors and primary intracranial tumors. 4) Inactive cancer is defined as a patient with a history of cancer who has undergone surgery more than 5 years prior to the onset of this acute stroke and has no symptoms or signs of recurrence. 5) Patients with cerebral hemorrhage, encephalitis, multiple sclerosis or other central nervous system diseases. 6) Patients with severe heart, liver, lung and renal insufficiency.

#### 4.1.2 Data collection

**FIGURE 1:**
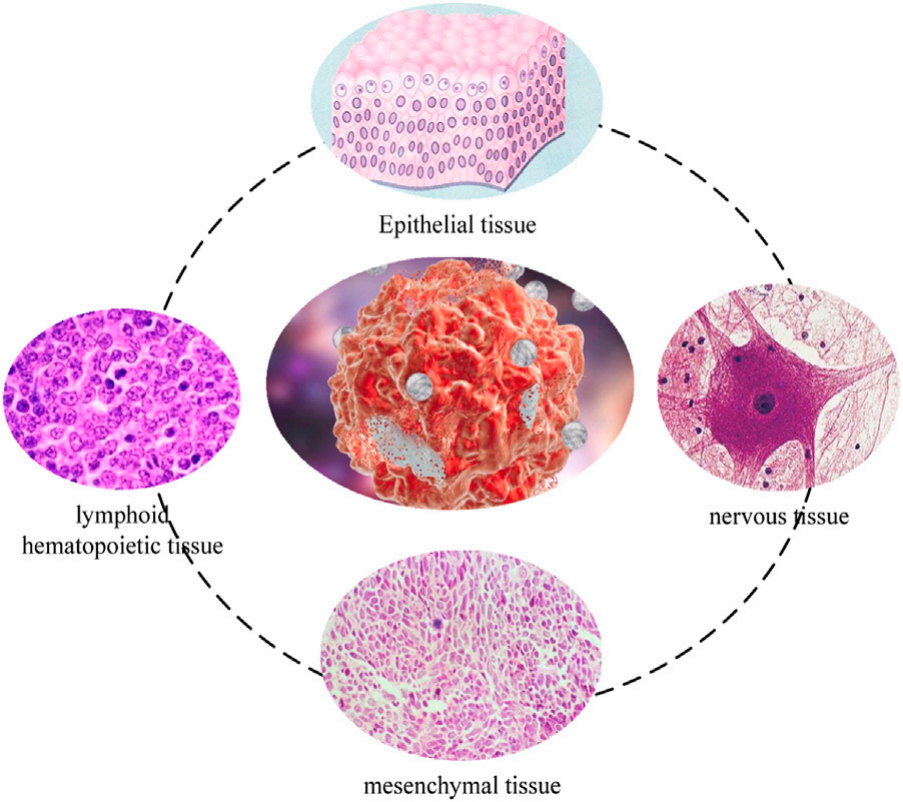
Classification of malignant tumors.

Clinical baseline data were collected for all patients, including patient age, sex, and time from cancer diagnosis to stroke event. This article documents traditional vascular risk factors for stroke, including hypertension, diabetes, hyperlipidemia, atrial fibrillation, smoking, and history of ischemic stroke. The patient’s laboratory tests have coagulation parameters D-dimer, fibrinogen degradation products, and fibrinogen values. We collected data on imaging studies of patients. Generally speaking, malignant tumors include many types, and the main sources include carcinoma of epithelial tissue, mesenchymal tissue source, lymphoid hematopoietic tissue source, neural tissue source and other tissue sources. The classification of malignant tumors is shown in Figure 1.

#### 4.1.3 Statistical analysis

Statistical analysis software SPSS22.0 was used for statistical analysis in this study. Individual factors of *p* < 0.1 have been incorporated into stepwise multiple accounting regression to obtain a complete predictive model. Then, the ROC curve analysis was used to compare the prediction performance of D_D, FDP, Fib and the comprehensive prediction model, and Kappa test was carried out between D-D, the comprehensive prediction model and the real results, and the test level was a = 0.05.

### 4.2 Clinical characteristics of patients with cancer-related acute ischemic cerebral infarction

**FIGURE 2:**
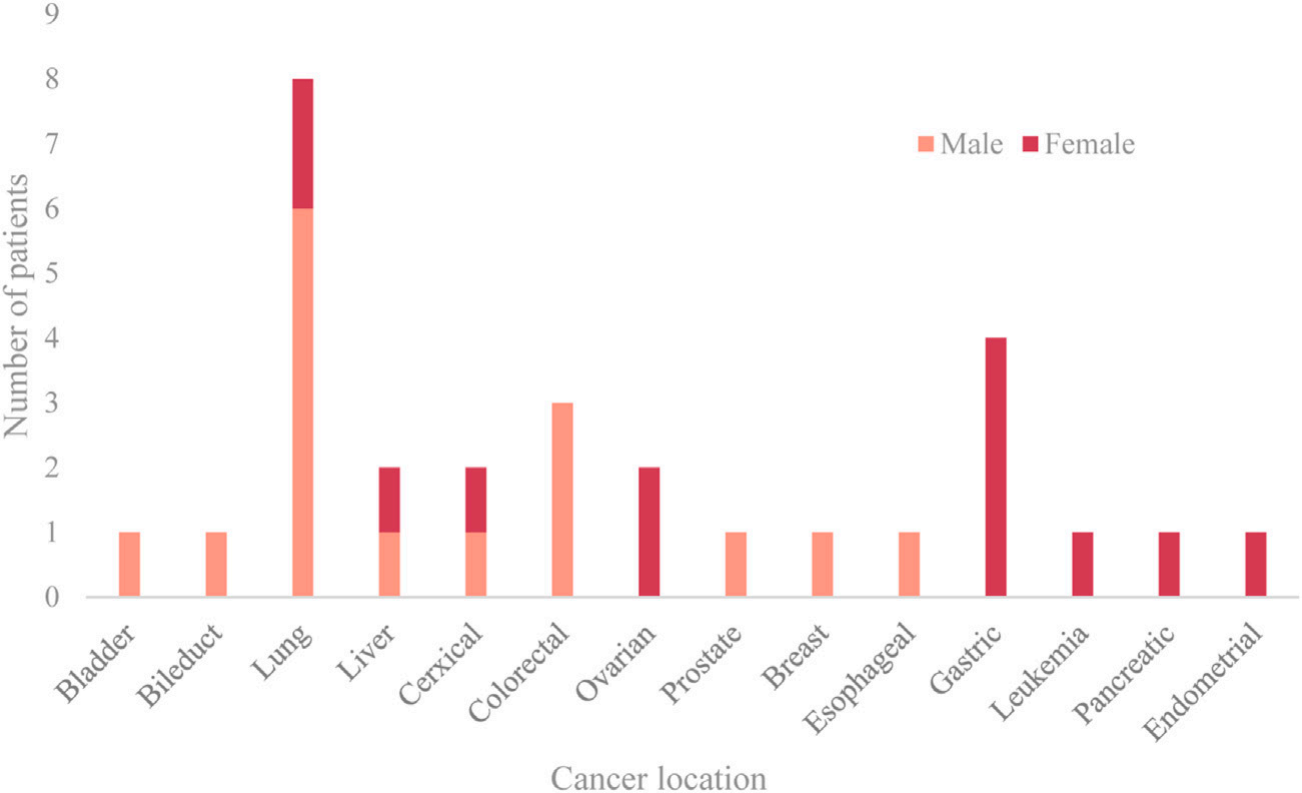
Location and sex ratio of primary cancer.

Lung cancer, gastrointestinal cancers, and cancers of the female reproductive system are the three most prevalent cancers. The median interval between the diagnosis of malignancy and the beginning of the stroke was 8.5 months. Acute cerebral infarction was the first presentation in four patients (14.3%), and cancer (ovarian cancer, lung cancer) was subsequently discovered during the neurology hospitalization. From the time of the cancer diagnosis until the commencement of the ischemia infarction, the distribution of time was as follows: 14.3% in 3, 14.3% in 3–6, 25.0% in 6–12, 25.0% in 12–24, and 7.1% in >24 months. The prevalence of risk factors, such as hypertension, diabetes, atrial fibrillation, hyperlipidemia, and smoking, is lower in the experimental group compared to the control group. While the value of fibrinogen was not substantially different, the D-dimer and FDP values of the cancer group were greater than those of the control group. The location and sex ratio of primary cancer are shown in Figure 2.

**FIGURE 3:**
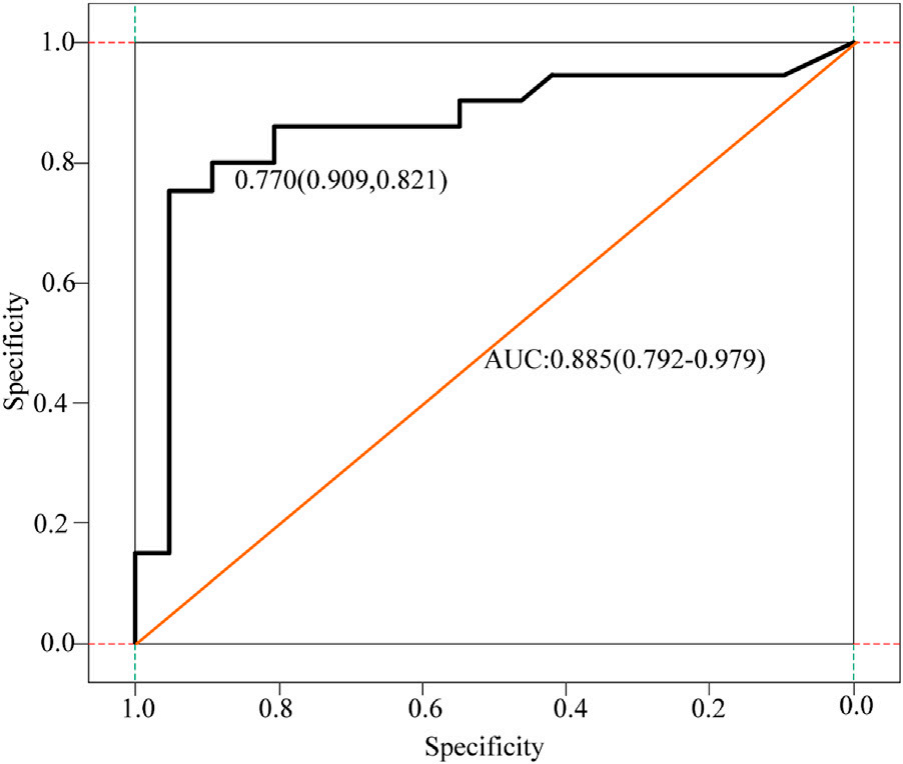
Receiver operating curves for cancer and non-cancer patients with stroke.

The D-dimer test results of a few patients (3 cases in the cancer group) exceeded the upper limit of hospital’s detection system. The receiver operating characteristic curve (ROC) analysis showed that the cut-off value of D-dimer to distinguish cerebral infarction patients with or without malignancy was 0.770 μg/ml. In the control group, 12.1% had values higher than the upper limit of the normal reference value of DD, and only two patients had DD values > 0.770 μg/ml. Besides, 17.9% of the patients in the cancer group had DD values < 0.770 μg/ml, and these 17.9% of patients also had DD values within the normal reference range. Figure 3 displays receiver operating curves for stroke patients with and without malignancy.

**FIGURE 4:**
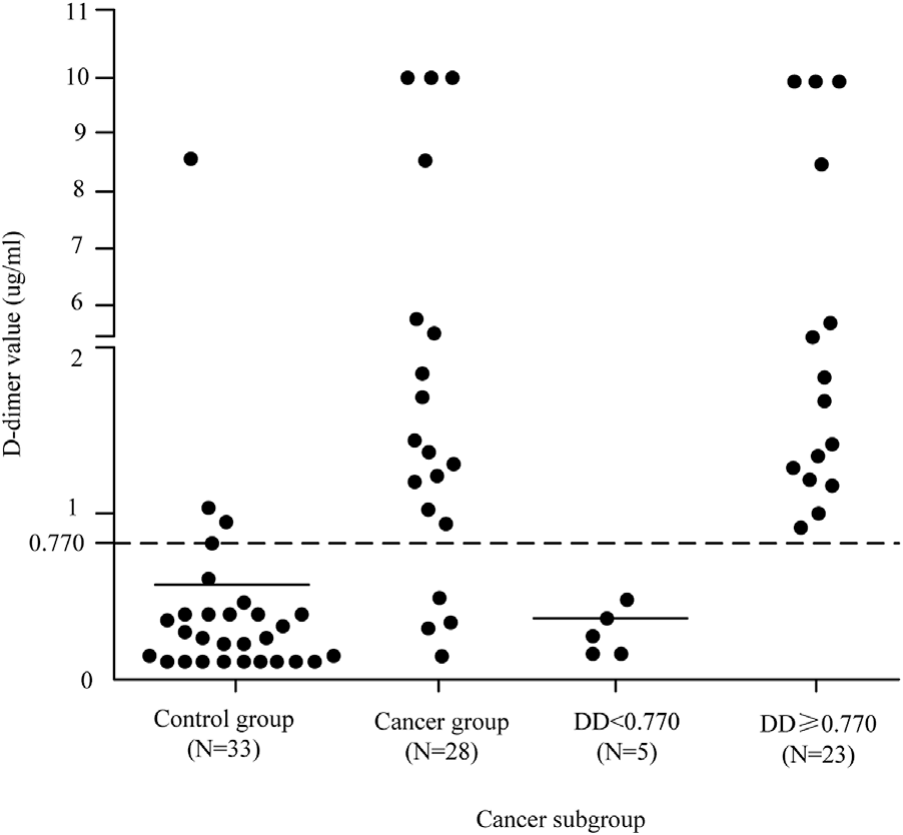
D-dimer value distribution patterns in each patient subgroup.

As can be seen from the analysis in Figure 3, the cancer group was divided into two subgroups with the value of 0.770 μg/ml as the critical value, D-dimer <0.770 μg/ml and D-dimer ≥0.770 μg/ml. The pattern of D-dimer values distribution in each subgroup of individuals with acute ischemic cerebral infarction is shown in Figure 4.

As can be seen from Figure 4, the D-dimer values in the cancer group were scattered, and when D-dimer was <0.770 μg/ml, the distribution matched that of the control group quite well.

### 4.3 Related risk factors in patients with malignant tumor-related cerebral infarction

The analysis results showed that plasma D-dimer (OR = 1.504, 95%CI:0.996–2.272, *p* = 0.052) and fibrin (ogen) degradation products (OR = 3.680, 95%CI:1.635–8.281, *p* = 0.002) level, and acute multiple cerebral infarction (OR = 13.099, 95%CI: 1.569–109.364, *p* = 0.018) were independent risk factors for acute ischemic cerebral infarction in patients with malignant tumors. The stepwise forward multivariate logistic regression analysis is shown in Table 1.

**TABLE 1:** Stepwise forward multivariate logistic regression analysis.

Variable	Estimate	Se	z	Wald	p	Or (95 CI%)
(Intercept)	−4.107	1.122	−3.661	13.404	0	0.016 (0.002, 0.148)
DD (u g/ml)	0.408	0.21	1.941	3.767	0.052	1.504 (0.996, 2.272)
FDP (ug/ml)	1.303	0.414	3.148	9.909	0.002	3.680 (1.635, 8.281)
AMBIs	2.573	1.083	2.376	5.645	0.018	13.099 (1569,109.364)

**FIGURE 5:**
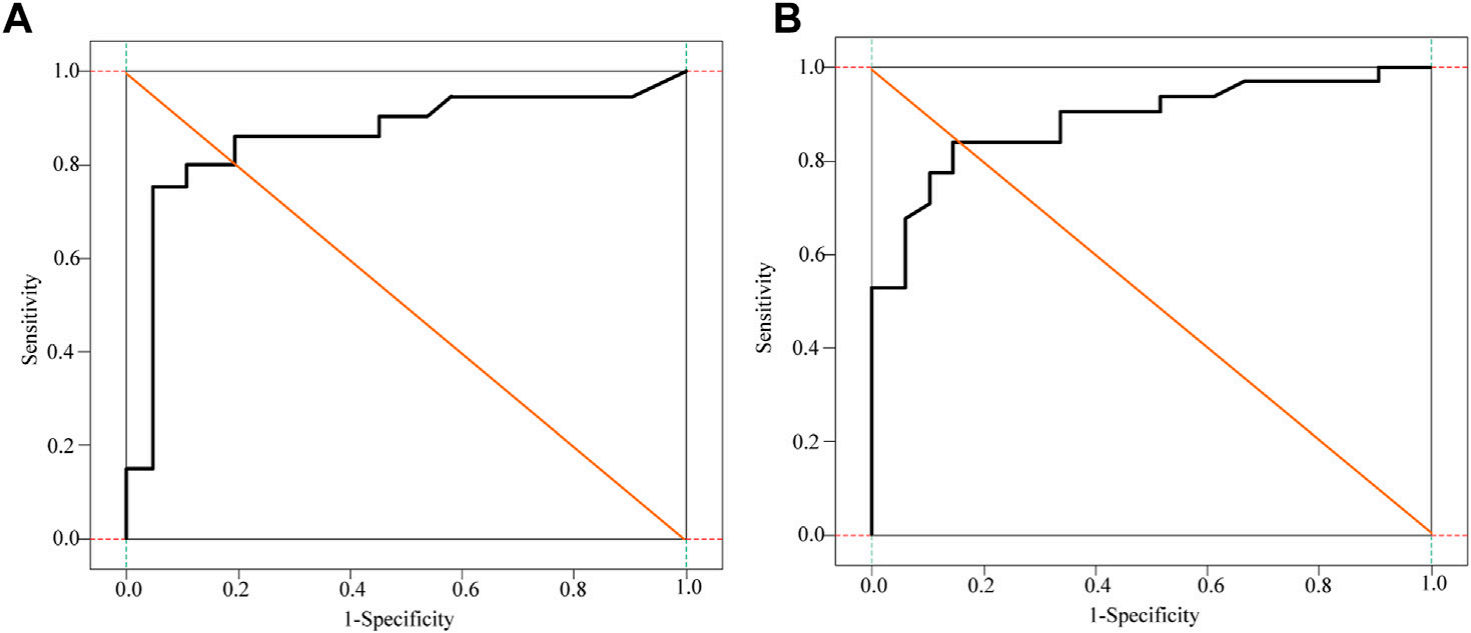
Subject D-D dimer and FDP curve analysis of patients. **(A)** D-D dimer curve **(B)** FDP curve.

**FIGURE 6:**
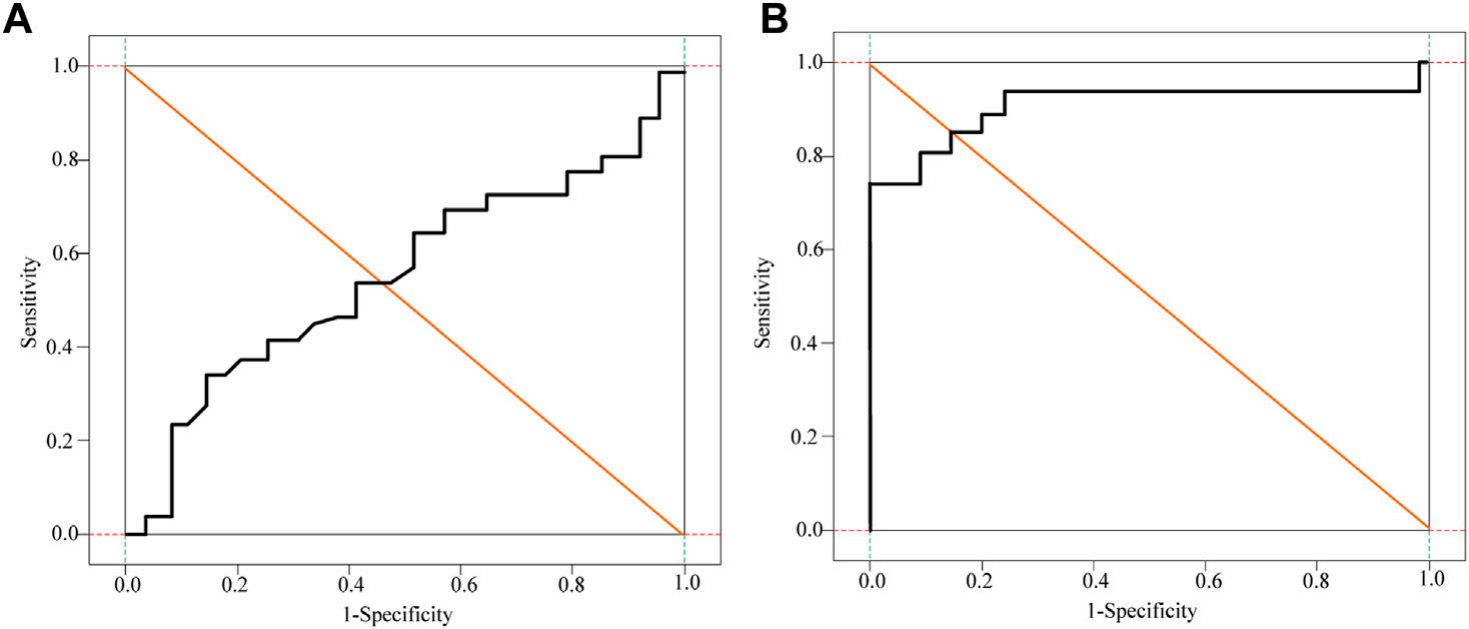
Subject Fib and Logistic Regression curve analysis of patients. **(A)** Fib curve **(B)** Logistic Regression curve.

It can be seen from Table 1 that a comprehensive prediction model was obtained based on the CAIS-related factors screened out in multivariate logistic regression, and the risk index of CAIS in the patients in this study was calculated according to the prediction model. The DD, FDP, Fib and comprehensive prediction models are analyzed and compared by drawing the ROC curve, as shown in Figure 5 and Figure 6.

It can be seen from Figure 5 and Figure 6 that DD, FDP and comprehensive prediction models all have high diagnostic value for malignant tumor-related cerebral infarction. However, the AUC of the comprehensive prediction model was 0.925, which was significantly higher than the AUC area predicted by other risk factors alone, and the predicted cut-off value was 0.636. The Kappa test was used to analyze the consistency of D-dimer levels, the comprehensive prediction model and the real results. The results of the Kappa consistency test are shown in Table 2.

**TABLE 2:** Kappa consistency test.

	Total (n = 61)	Control group (n = 33)	Cancer group (n = 28)	Kappa (95%C)	P
D-dimer value					
<0.770	35 (57.38)	30 (90.91)	5 (17.86)	0.734 (0.554–0.899)	<0.001
20.770	26 (42.62)	3 (9.09)	23 (82.14)		
Comprehensive forecast				0.799 (0.632–0.932)	<0.001
<0.636	39 (63.93)	33 (100.00)	6 (21.43)		
20.636	22 (36.07)	0 (0.00)	22 (78.57)		

It can be seen from Table 2 that D-D≥0.770 μg/ml (Kappa = 0.734, *p* < 0.001) and the comprehensive prediction model (Kappa = 0.799, *p* < 0.001) are in good agreement with the real results. However, the comprehensive prediction model has a high degree of consistency. Therefore, compared with DD ≥ 0.770 μg/ml for the diagnosis of malignant tumor-related cerebral infarction, the prediction effect of the comprehensive prediction model may be better.

## 5 CT imaging features

### 5.1 CT imaging features of 83 patients with G1 pNENs and G2 pNENs

The tumor size of G2 pancreatic neuroendocrine tumor was significantly different from that of G1 pancreatic neuroendocrine tumor (47.4 + 28.9 VS. 28.6 + 17.7, *p* < 0.01). The CT imaging capabilities of G1 and G2pNEN showed that the largest tumor diameter was larger in G2, and the rest were size, number, border, shape, cystic degeneration, homogeneous enlargement, pancreatic duct enlargement, calcification, peripheral lymph node metastasis, and distant metastasis. The difference was not statistically significant.

### 5.2 The clinical value of CT absolute and relative enhancement values in predicting G1 and G2 pathological grades

To further clarify the clinical value of CT-related imaging functions in determining abnormal G1 and G2 scores, ROC curves were used to identify and analyze the absolute and relative CT magnification values of patients with G1 and G2 pNETs. The ROC analysis results of CT absolute and relative enhancement in the clinical diagnosis of G1 and G2 pNENs are shown in Table 3.

**TABLE 3:** ROC analysis results of CT absolute and relative enhancement in clinical diagnosis of G1 and G2 pNENs.

Degree of reinforcement (CT value)	AUC	95%CI	Specificity	Sensitivity	Cut-off
Absolute arterial phase	0.775	0.648–0.901	0.621	0.926	69.55
Portal phase absolute	0.811	0.700–0.922	0.655	0.889	84.20
Absolute delay	0.803	0.687–0.918	0.926	0.586	55.85
Arterial phase relative	0.692	0.553–0.831	0.483	0.889	12.85
Portal phase relative	0.760	0.633–0.887	0.517	0.926	11.15

**FIGURE 7:**
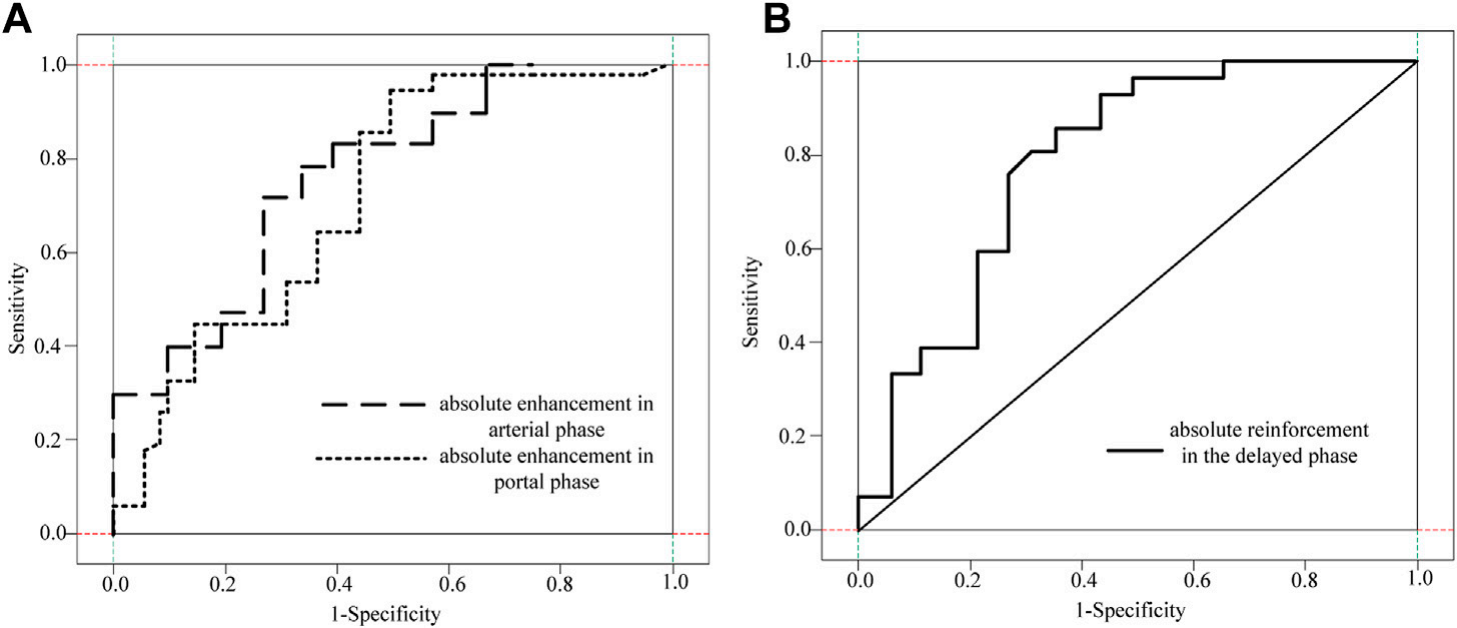
ROC curve analysis of three different absolute enhancement degrees. **(A)** Absolute enhancement in arterial and portal phases **(B)** Absolute enhancement in delayed phase.

**FIGURE 8:**
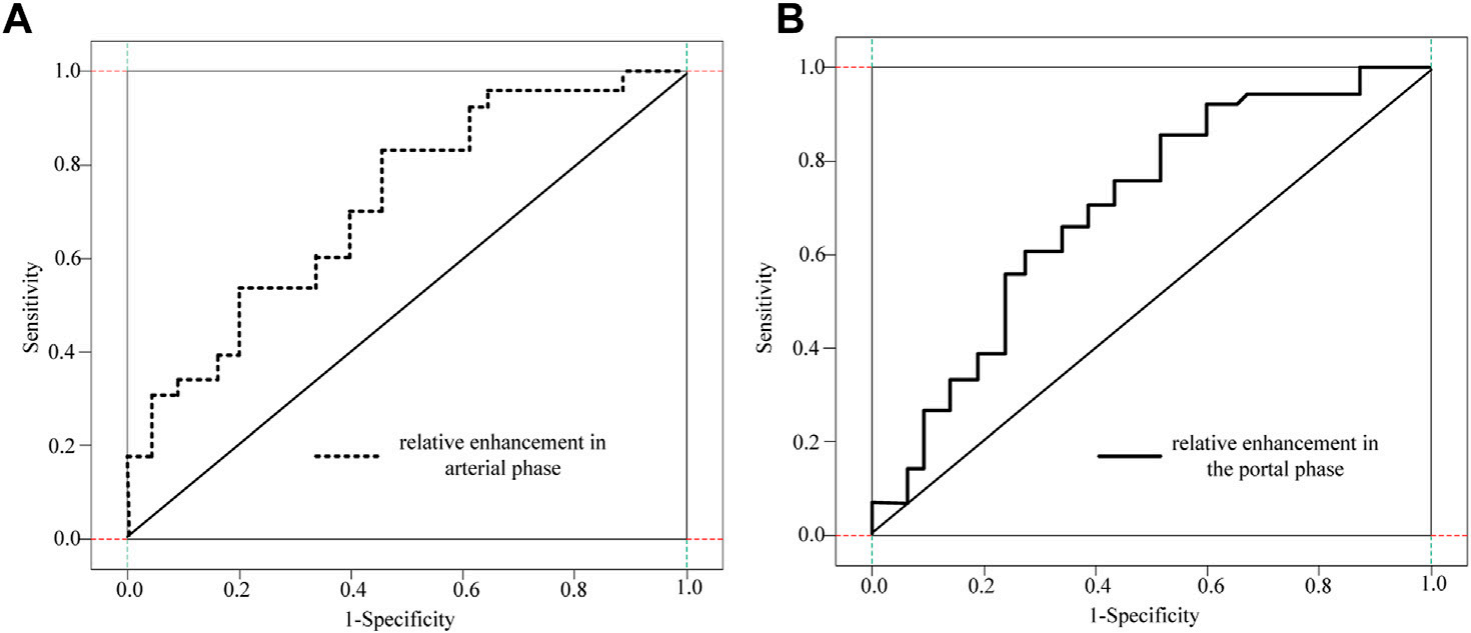
ROC curve analysis of two different relative enhancement degrees. **(A)** Relative enhancement degree in arterial phase **(B)** Relative enhancement degree in portal venous phase.

The ROC curve analysis of different absolute enhancement degrees and the ROC curve analysis of two different relative enhancement degrees are shown in Figure 7 and Figure 8.

As shown in Figure 7 and Figure 8, the absolute and relative CT magnification values can effectively distinguish the G1 and G2 spin pNEN patients from the area under the absolute magnification ROC (AUC) curve. The portal phase value was 0.811 (95% CI 0.700–0.922), the specificity was 0.811 (95% CI 0.700–0.922) 0.655, and the sensitivity was 0.889.

**FIGURE 9:**
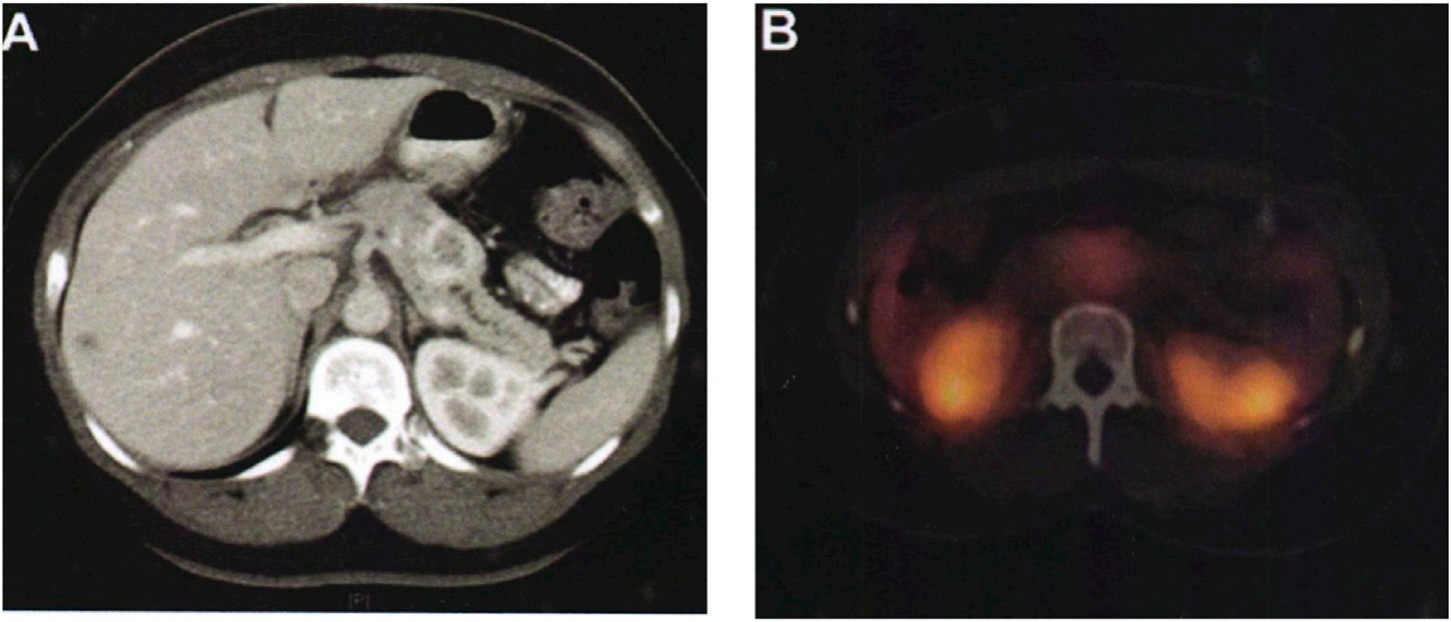
Comparison of patients before and after surgery. **(A)** The pancreas tissue of the patient before surgery. **(B)** The pancreas tissue of the patient after surgery.

Multi-slice computed tomography (CT) or magnetic resonance imaging (MRI) of the pancreas is still the preferred method for evaluating the imaging manifestations of neuroendocrine tumors and the extent of tumor invasion. On multiphase CT, non-functional pancreatic neuroendocrine tumors appear as well-defined peripheral hypervascular lesions, which are most clearly visualized in the end arterial and portal phases. The extent of local vascular invasion can be used as an indicator for assessing resectability. The sensitivity of CT examination is 63%–82%, and the specificity is 83%–100%, which varies according to its size. The sensitivity for detecting liver metastases was 82% and the specificity was 92%. About 10% of non-functional pancreatic neuroendocrine tumors are found as cystic lesions of the pancreas, with a misdiagnosis rate of about 43%. MRI improves tissue contrast in assessing the pancreas and liver. Its sensitivity is 85%–100%, the specificity is 75%–100%, the average detection rate of non-functioning neuroendocrine tumors is 73%, and the average detection rate of liver metastases is 82%. Preoperative examinations also included magnetic resonance cholangiopancreatography (MRCP). MRI is most useful when monitoring or confirming liver metastases. The comparison of the patients before and after surgery is shown in Figure 9.

As shown in Figure 9A, a 36-year-old female patient, who was unfit for contrast-enhanced CT examination because of abdominal pain, showed typical imaging manifestations of pancreatic neuroendocrine tumors with marked enhancement in the arterial phase. The hypodense foci in the right hepatic lobe were not visualized on octreotide scanning, and were assessed as hepatic cysts by MRI. Pancreatic lesions were removed by laparoscopic pancreatic body and tail + splenectomy. There was no recurrence or metastasis in the follow-up 2 years after operation. Figure 9B shows a preoperative octreotide scan of the same patient showing typical features of a non-functional pancreatic neuroendocrine tumor with mildly elevated octreotide uptake.

In addition to traditional imaging, there are two nuclear medicine examination methods: somatostatin receptor scintigraphy (SRS) and positron emission tomography (PET). The somatostatin receptor family (SSTRs) includes five subtypes. That is, SSTR1 is highly expressed in many pancreatic neuroendocrine tumors, especially SSTR-2. It can therefore be visualized by radionuclide-labeled somatostatin receptor analogs (octreotides). SRS is often used to locate, stage, check for metastases, monitor recurrence, and evaluate response to systemic therapy in non-functional pancreatic neuroendocrine tumors. There are no data to support these tests to provide more valuable information than CT, MRI. In addition, SRS examination is expensive, which will increase the financial burden of patients.

## 6 Conclusion

In this study, the clinical and pathological characteristics of 266 patients with pancreatic neuroendocrine tumors were compared and analyzed, and it was found that patients with GI grade pancreatic neuroendocrine tumors and G2 and G3 pancreatic neuroendocrine tumors had obvious lymphadenopathy. The difference in distant metastases was statistically significant, but the extent of gastrointestinal and pancreatic neuroendocrine tumors showed only statistically significant differences in tumor diameter on computed tomography. This analysis was performed for the following reasons: computed tomography scans are more reliable and objective for larger diameter tumors, but standard CT scans are sufficiently objective for distant metastases, lymph node metastases, and invasive tumor growth. At the same time, 83 cases are relatively small, and reducing the sample size will have a certain impact on the results. A retrospective analysis of the preoperative CT image data of 56 patients with G1 and G2pNEN showed that the CT image features of GI and G2 were significantly different in CT values. The absolute and relative amplitudes of the arterial and portal phases were significantly higher than those of G2-grade patients and had important diagnostic implications in distinguishing G1-grade and G2pNEN patients. Of course, this study also has some shortcomings. The first study was designed as a retrospective analysis and had some selection bias. Second, because this was a retrospective study, the criteria for determining feature expression on specific CT images were inconsistent and biased. Third, due to the small number of G3 pNEN cases, this study should only include imaging features of G1 and G2 patients, and further studies are needed to analyze G3 patients.

## Data Availability

The original contributions presented in the study are included in the article/Supplementary Material, further inquiries can be directed to the corresponding author.
